# Administration of Adult Human Bone Marrow‐Derived, Cultured, Pooled, Allogeneic Mesenchymal Stromal Cells in Critical Limb Ischemia Due to Buerger’s Disease: Phase II Study Report Suggests Clinical Efficacy

**DOI:** 10.5966/sctm.2016-0237

**Published:** 2016-10-05

**Authors:** Pawan K. Gupta, Murali Krishna, Anoop Chullikana, Sanjay Desai, Rajkumar Murugesan, Santanu Dutta, Uday Sarkar, Radhakrishnan Raju, Anita Dhar, Rajiv Parakh, Lakshmanan Jeyaseelan, Pachaiyappan Viswanathan, Prasanth Kulapurathu Vellotare, Raviraja N. Seetharam, Charan Thej, Mathiyazhagan Rengasamy, Sudha Balasubramanian, Anish S. Majumdar

**Affiliations:** ^1^ Stempeutics Research, Bangalore, India; ^2^ Department of Vascular Surgery, Sri Jayadeva Institute of Cardiovascular Sciences, Bangalore, India; ^3^ Department of Vascular Surgery, MS Ramaiah Medical College & Hospitals, Bangalore, India; ^4^ Department of Vascular Surgery, SRM Medical College, Chennai, India; ^5^ Department of Cardiovascular Surgery, Nightingale Hospital, Kolkata, India; ^6^ Department of Cardiovascular Surgery, Health Point Hospital, Kolkata, India; ^7^ Department of Vascular Surgery, Sri Ramachandra Medical College, Chennai, India; ^8^ Department of Surgical Disciplines, All India Institute of Medical Sciences, New Delhi, India; ^9^ Division of Peripheral Vascular and Endovascular Sciences, Medanta‐The Medicity, Gurgaon, Haryana, India; ^10^ Department of Biostatistics, Christian Medical College, Vellore, India; ^11^ Manipal University, Manipal, India

**Keywords:** Stempeucel, Critical limb ischemia, Buerger’s disease, Bone marrow, Mesenchymal stromal cells

## Abstract

Critical limb ischemia (CLI) due to Buerger’s disease is a major unmet medical need with a high incidence of morbidity. This phase II, prospective, nonrandomized, open‐label, multicentric, dose‐ranging study was conducted to assess the efficacy and safety of i.m. injection of adult human bone marrow‐derived, cultured, pooled, allogeneic mesenchymal stromal cells (BMMSC) in CLI due to Buerger’s disease. Patients were allocated to three groups: 1 and 2 million cells/kg body weight (36 patients each) and standard of care (SOC) (18 patients). BMMSCs were administered as 40–60 injections in the calf muscle and locally, around the ulcer. Most patients were young (age range, 38–42 years) and ex‐smokers, and all patients had at least one ulcer. Both the primary endpoints—reduction in rest pain (0.3 units per month [SE, 0.13]) and healing of ulcers (11% decrease in size per month [SE, 0.05])—were significantly better in the group receiving 2 million cells/kg body weight than in the SOC arm. Improvement in secondary endpoints, such as ankle brachial pressure index (0.03 [SE, 0.01] unit increase per month) and total walking distance (1.03 [SE, 0.02] times higher per month), were also significant in the group receiving 2 million cells/kg as compared with the SOC arm. Adverse events reported were remotely related or unrelated to BMMSCs. In conclusion, i.m. administration of BMMSC at a dose of 2 million cells/kg showed clinical benefit and may be the best regimen in patients with CLI due to Buerger’s disease. However, further randomized controlled trials are required to confirm the most appropriate dose. Stem Cells Translational Medicine *2017;6:689–699*

**Significance Statement**

Critical limb ischemia (CLI) due to Buerger’s disease presents a major unmet medical need. The limited therapeutic options lead to increased morbidity and mortality. This study showed that use of adult human bone marrow‐derived, cultured, pooled, allogeneic mesenchymal stromal cells is safe and efficacious when the cells are injected intramuscularly at a dose of 2 million cells/kg body weight in patients with CLI. Rest pain and ulcer healing significantly improved in most patients. This regimen may be a novel therapeutic option for Buerger’s disease.

## Introduction

Thromboangiitis obliterans, also known as Buerger’s disease, is a nonatherosclerotic, segmental inflammatory disease that most commonly affects the small and medium‐sized arteries and veins in the upper and lower extremities [1]. The disease progresses to critical limb ischemia (CLI), manifested clinically as rest pain, incurable ulceration, gangrene, and toe or limb loss. Because the possibility of vascular bypass is usually small as a result of diffuse segmental involvement and the distal nature of the disease [2], the consequence of the disease in the more advanced stage may be limb amputation, especially of the lower extremities [3]. Consequently, there is increasing ongoing research on the use of additional interventions for therapeutic angiogenesis, such as cell‐based therapy [4, 5, 6, 7, 8, 9], gene therapy [10,11] and immunoabsorption therapy [12] in these “no option” patients with CLI.

Our previous report [13] showed that use of adult human bone marrow‐derived, cultured, pooled, allogeneic mesenchymal stromal cells (BMMSCs; Stempeucel, Stempeutics Research, Bangalore, India, http://www.stempeutics.com ) was safe when injected via the i.m. route at a dose of 2 million cells/kg body weight in patients with CLI. The probable mechanism of action of Stempeucel is likely due to a combinatorial effect of anti‐inflammatory and proangiogenic activity governed by paracrine function or by directly producing the cytokines and growth factors at the site of inflammation and ulcer location.

Most Buerger’s disease patients in India are relatively young male smokers in families belonging to low socioeconomic strata [14]; this causes economic morbidity in the family and society alike. Studies have shown that approximately 0.4 million people in India are affected by CLI due to Buerger’s disease [15], indicating the gravity of the disease in this country. Given the limitation of the current therapies and high rate of amputations and mortality, the quality of life of these patients is compromised and is likened to that of patients with terminal cancer [16]. Given the urgent need for an alternative therapy in these “no‐option” patients, we conducted a study to determine the efficacy and safety of Stempeucel in patients with CLI due to Buerger’s disease.

## Materials and Methods

### Preclinical Studies

A series of preclinical safety/toxicological studies were performed in rodents (rat) and nonrodent (rabbit) species; these included acute, subchronic toxicity, and teratogenicity studies. The cells were administered both by i.v. and i.m. routes up to a maximum of 252 × 10^6^ cells/kg body weight. Tumorigenicity studies were conducted in SCID mice by using a dose of 10 × 10^6^ cells/kg body weight, which were administered by i.m. and s.c. routes. Animals were observed for 6 months, and no signs of tumor development was seen. Preclinical efficacy was assessed in BALB/c nude mice (OlaHsd‐Fox1nu) aged 10–12 weeks; after the mice were anesthetized, the left femoral artery was ligated and resected [17]. Stempeucel was administered at a dose of 5.0 × 10^6^ cells/kg body weight intramuscularly in the adductor muscle; five injections total were given, each constituting of 10‐µl volume within 2 hours of arterial ligation (*n* = 10). Animals receiving sham treatment (*n* = 3) were used as control, and animals injected with PlasmaLyte A (*n* = 10; Baxter, Deerfield, IL, http://www.baxter.com) served as vehicle control. Animals were followed up for 28 days.

### Study Design

This prospective, nonrandomized, open‐label, multicentric, dose‐ranging, phase II study assessed the efficacy and safety of i.m. administration of Stempeucel in patients with CLI due to Buerger’s disease. This study compared patients with lower‐extremity CLI due to Buerger's receiving i.m. injections of pooled allogeneic BMMSCs suspended in multiple electrolyte solutions (PlasmaLyte A) with patients receiving the standard of care (SOC) alone. After we obtained informed consent, 90 patients were enrolled in this dose‐finding study into three study groups in a sequential manner: a group receiving 1 million cells/kg body weight (first 36 patients), a group receiving 2 million cells/kg body weight (next 36 patients), and the SOC group (last 18 patients) during a period of 24 months. However, in between, if any consenting patient declined to be enrolled in the stem cell arm, they were enrolled in the control arm. The study was sponsored by Stempeutics Research. The protocol was approved by Central Drugs Standard Control Organization (Indian Food and Drug Administration) and the institutional ethical committees of all 11 participating hospitals in India. Informed consent was obtained from all the participating patients before they were included in the phase II study. An independent data safety monitoring board (DSMB) was established, which assessed the progress of this study and reviewed the safety of the clinical data (all serious adverse events and available adverse event data at the time of scheduled DSMB meetings) at periodic intervals according to the DSMB charter specially developed for this study. The study was registered on the National Institutes of Health and Clinical Trials Registry‐India [CTRI] website (ClinicalTrials.gov identifier: NCT01484574; CTRI number: CTRI/2011/11/002107).

### Inclusion/Exclusion Criteria

**Figure 1:**
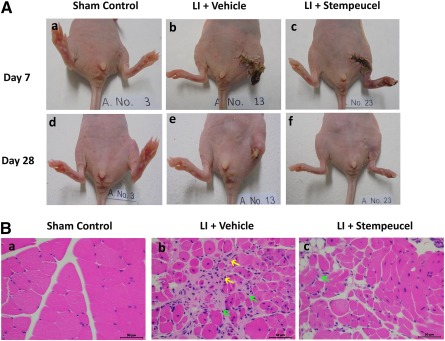
Stempeucel treatment ameliorates ischemia‐induced limb necrosis and limb salvage in mice. **(A):** Representative photographs of sham control **(Aa, Ad)**, vehicle‐treated limb ischemia **(Ab, Ae),** and Stempeucel‐treated **(Ac, Af)** LI on day 7 and day 28 after cell treatment. Stempeucel treatment significantly improved limb salvage in comparison with vehicle. **(Ba, Bb, Bc):** Hematoxylin and eosin‐stained sections of adductor muscle show severe necrosis (yellow arrows) and infiltration of inflammatory cells (green arrows), which were markedly reduced in the Stempeucel‐treated animals **(Bc)**. Original magnification, ×40; scale bars = 50 µm. Abbreviation: LI, limb ischemia.

Patients with established CLI due to Buerger’s disease who had not responded to or were not eligible for a revascularization procedure were included in the study (Table 1). Information on the total numbers of patients screened, allocated in each arm, followed up, and analyzed in the trial is given in Figure 1 (diagram as per the Consolidated Standards of Reporting Trials flowchart). The visits were scheduled on day −10 (screening visit), day 0 (injection of Stempeucel), and follow‐up visits on day 7 ± 3 days, month 1 ± 3 days, month 3 ± 3 days, and month 6 ± 7 days. All 90 enrolled patients in the two dose groups (1 and 2 million cells/kg body weight) and the SOC group will be followed until completion of the 24‐month efficacy and safety endpoints.

**Table 1:** Inclusion and exclusion criteria for study enrollment

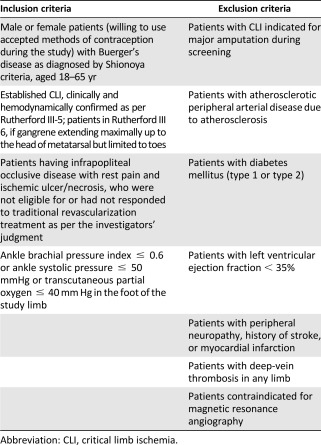

### Preparation of Stempeucel and Intramuscular Injection

Stempeucel, a pooled, allogeneic BMMSC product, was obtained from bone marrow aspirates from consenting donors who were not human leukocyte antigen matched to the recipients. Healthy bone marrow donors were tested according to 21 Code of Federal Regulations 1271, U.S. Food and Drug Administration donor suitability and Indian Council of Medical Research guidance for healthy bone marrow donor screening. BMMSCs were obtained from bone marrow samples of healthy donors aged 18–40 years after they provided informed consent. The protocol was approved by the institutional ethics committee.

Briefly, 60 ml of bone marrow aspirate was diluted (1:1) with knockout DMEM (Thermo Fisher Scientific Life Sciences, Waltham, MA, http://www.thermofisher.com), and was centrifuged at 1,800*g* for 10 minutes to remove the anticoagulant. Bone marrow mononuclear cells (BMMNCs) were isolated by the density gradient centrifugation (1.077 g/ml) method as described elsewhere [17]. BMMSCs were isolated by plastic adherence from the donor’s BMMNCs and cultured until passage 1. A donor master cell bank constituted from MSCs from an individual BM sample was created and maintained under cryopreserved conditions. Subsequently, a working cell bank (WCB) was prepared by combining MSCs from two or more donors and cryopreserved for manufacturing Stempeucel and further expanding the pooled WCB for additional passages for manufacturing Stempeucel (U.S. patent number 8956862, dated February 17, 2015). For the work described in this article, pooled BMMSCs from three different individual donors were cultured, harvested, and characterized at passage 5 and cryopreserved in liquid nitrogen as the final product [18]. Criteria for Stempeucel specification release are the same as given in our earlier report [13].

Expanded Stempeucel (100 million ± 10%/200 million ± 10%) were stored in 15 ml of PlasmaLyte A (multiple electrolytes injection, type 1, U.S. Pharmacopeia) containing 5% human serum albumin (Baxter) and 10% dimethyl sulfoxide (Sigma‐Aldrich, Irvine, United Kingdom, https://www.sigmaaldrich.com) in a Cryocyte bag (MacoPharma, Mouvaux, France, http://www.macopharma.com). The cryopreserved cells were thawed and resuspended in 35 ml of PlasmaLyte A, resulting in 50 ml of final cell suspension. Stempeucel formulation consisted of 2.0 × 10^6^/4.0 × 10^6^ Stempeucels per ml and 1.6% human serum albumin with 3.33% dimethyl sulfoxide in multiple electrolytes injection (PlasmaLyte A) solution. Stempeucel was administered under epidural/general anesthesia, and cardiorespiratory monitoring was performed in all patients. The reconstituted volume of suspension (1 million/2 million/0.5 ml/kg body weight) was injected intramuscularly into the gastrocnemius muscle of the ischemic lower limb (40–60 sites, distributed in an area of 10 cm × 6 cm, 1–1.5 cm deep, and 0.5–1.0 ml of Stempeucel per site) in 3‐ml syringes. In addition, 2 ml (4 million cells [for the 1 million/kg dose] or 8 million cells [for the 2 million/kg dose]) of the reconstituted Stempeucel was also injected intramuscularly around the ulcer (4–6 sites; 0.3–0.5 ml of Stempeucel per site). The patients remained in the hospital under supervision for at least 24 hours after Stempeucel administration. On discharge, patients were given standard of care for CLI as per the investigator’s discretion. This included but was not limited to analgesics, antiplatelets, phosphodiesterase inhibitors, statins, and wound care. The patients enrolled into the control arm were given standard of care only as per investigators' discretion.

### Study Endpoints

#### Efficacy Evaluation

Primary efficacy measures included relief of rest pain (measured by visual analog scale (VAS) that ranged from 0 for no pain and 10 for worst pain) and ulcer healing (measured by Visitrak, Smith & Nephew, London, UK, http://www.smith‐nephew.com). Ulcer healing was assessed by an independent physician and was photographically documented to avoid bias. Visitrak includes a tracing film (Visitrak grid) for obtaining the outline of the ulcer. The film placed on a Visitrak portable digital tablet, and the outline of the ulcer is traced to obtain the ulcer area.

Secondary efficacy endpoints included increase in total walking distance (only in patients who could walk on a treadmill at 3.2 km/h with a 12% incline), increase in ankle brachial pressure index (ABPI; measured by using laser Doppler imaging), change in quality of life (QOL; measured by King’s College VascuQoL questionnaire), and evidence of increased collateral blood vessel formation by magnetic resonance angiography (MRA; performed at the time of inclusion and after 6‐month follow‐up). All other efficacy endpoints were evaluated at baseline and at 1‐month (visit 4), 3‐month (visit 5), and 6‐month (visit 6) follow‐up visits.

#### Safety Evaluation

Secondary safety measures included monitoring and recording of all adverse events (AEs) and assessment of electrocardiographic parameters, hematological and biochemical values, regular vital signs measurements, and physical examination.

### Data Collection

All data were recorded on electronic case record forms and verified by comparison with source documentation by third‐party medical monitors. Safety assessments were performed on the basis of frequency of AEs and clinically significant abnormal laboratory values.

### Statistical Analysis

#### Sample Size Calculation

Because this was a phase II dose‐finding study, its primary purpose was to establish the appropriate efficacious dose of Stempeucel. The planned sample size was based on 72 patients in the Stempeucel arm (36 patients in each dose group) and 18 patients in the control arm. This was calculated to detect a between‐group difference in change from baseline to 6 months of 0.47% in rest pain and 0.39% in ulcer healing (α = 0.05, two‐sided; 80% power). We also factored dropout rate of 10% and amputation rate of 30%.

#### Analysis of Data

The SAS software package, version 9.2 (SAS Institute Inc., Cary, NC, http://www.sas.com), was used for statistical evaluation. All data are presented as mean ± SE. AEs are summarized descriptively by total number of AEs for each treatment group by system organ class. The efficacy parameters were analyzed by using a generalized estimating equation (GEE) model with longitudinal analysis and chi‐square test as appropriate. A *p* value <.05 was considered to indicate a statistically significant difference.

## Results

### Preclinical Studies

Acute and subchronic toxicity studies showed that the maximum tolerated dose was ≥126 × 10^6^ cells/kg body weight in rats and ≥65.3 × 10^6^ cells/kg body weight in rabbits. Cells were also found to be nontumorigenic and nonteratogenic in immunocompromised (SCID mice) and immunocompetent animals, respectively [19]. The preclinical efficacy study showed that in the vehicle‐injected control group, 9 of 10 animals developed foot and ankle necrosis by day 3 and more than 70% of these animals sustained foot, ankle, or limb loss by day 7 (Fig. 1 Ab, 1Ae). Strikingly, 70% (7 of 10) of the animals receiving Stempeucel did not develop foot necrosis, although a few animals showed toe necrosis only (Fig. 1 Ac, 1Af; *p* < .01). No limb necrosis was observed in sham control animals (*n* = 3) (Fig. 1Aa, 1Ad). Hematoxylin and eosin staining of the adductor muscle of one representative animal from each group is shown (Fig. 1B). Extensive mononuclear cell infiltration and tissue necrosis were observed at 28 days in the vehicle‐injected animal (Fig. 1Bb). In contrast, Stempeucel‐treated animals showed significantly fewer infiltrating cells in the adductor muscle (Fig. 1Bc); no cellular infiltration was observed in the muscle section of the sham control animal (Fig. 1Ba).

### Patient Characteristics

**Figure 2:**
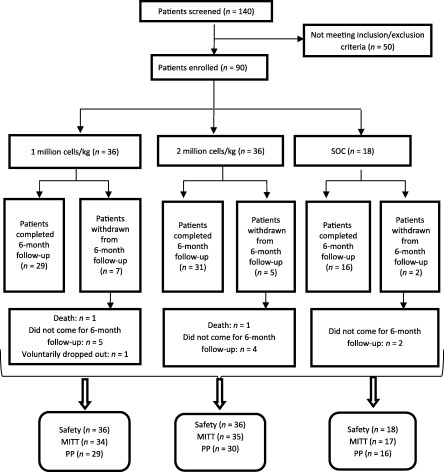
Total number of patients screened, allocated in each arm, followed up, and analyzed in the trial. Diagram as per the Consolidated Standards for Reporting Trials (CONSORT) flowchart. MITT population refers to all patients who received Stempeucel and had at least one rest‐pain assessment at visit 4. PP population refers to patients who had both the baseline and visit 6 rest‐pain assessments, with no major protocol deviation. Abbreviations: MITT, modified intention‐to‐treat; PP per protocol; SOC, standard of care.

The disposition of patients who were enrolled in and completed the study to 6‐months follow‐up visit is shown in Figure 2. Demographic and baseline patient data are listed in Table 2. Most patients were young (age range, 38–42 years) and ex‐smokers, and all patients had at least one ulcer.

**Table 2:** Demographic and baseline disease characteristics

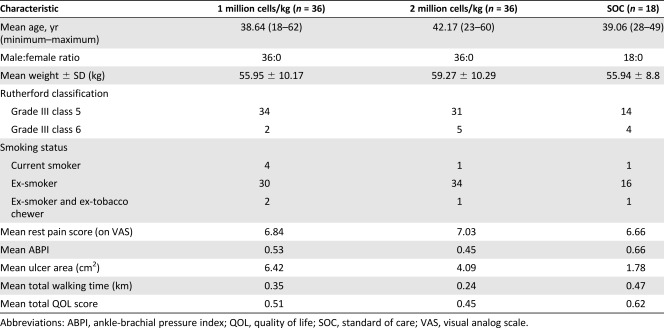

### Procedural Safety

No infection, bleeding, or other complications related to the microbiological condition of the cells were detected in any patient after administration of Stempeucel. Administration was well tolerated. No other procedure‐related complications, such as allergic reactions or local swelling, resulted from intramuscular injection of Stempeucel, suggesting that Stempeucel could be safely injected in patients with CLI due to Buerger’s disease.

### Safety Outcomes

The total number of adverse events, as classified by MedDRA Primary System Organ Class and Preferred Term system, recorded in the study was 47 (Table 3). The overall incidence of AEs did not differ among the three arms, with 19 AEs reported by 13 patients in the 1 million cells/kg group, 19 AEs in the 10 patients in the 2 million cells/kg group, and 9 AEs in 5 patients in the SOC group. All the AEs experienced in the study in the 1 million cells/kg group and the SOC group were treatment emergent adverse events (TEAEs). However, in the group receiving 2 million cells/kg, one amputation and one instance of debridement occurred in one patient each before Stempeucel administration. Skin ulcer was the most frequently observed TEAEs in the 1 million cells/kg group (6 events in 5 patients) and the SOC group (2 events in 2 patients). Gangrene was the most frequent TEAEs in the 2 million cells per group (4 events in 3 patients). All the TEAEs in the 3 arms of the study were assessed as remotely related or unrelated to Stempeucel; rather, they were considered to be related to the underlying condition. One event (pain at the site of administration) in 1 patient was assessed as probably related to Stempeucel. This event resolved within 1 month of follow‐up.

**Table 3:** Summary of adverse events, including common adverse events, in each group

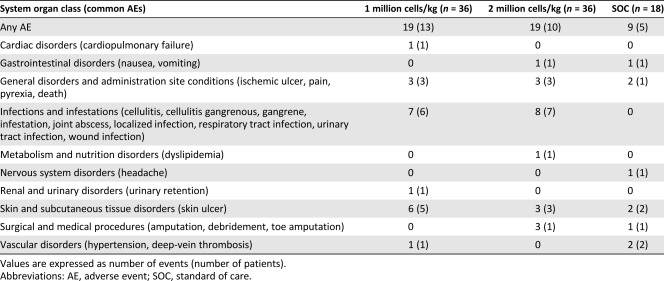

Two deaths were reported during the study period. One patient in the 1 million cells/kg group had cardiorespiratory failure following respiratory tract infection (patient ID: 5009), which resulted in death after 2 months of Stempeucel administration. However, the event was remotely related or unlikely to be related to the study drug. One patient in the 2 million cells/kg group died (patient ID: 4013) at home of unknown cause during the study period after 5 months of Stempeucel administration. The patient had vomiting, diarrhea, progression of gangrene, and significant swelling of both lower limbs, followed by fever; the patient was bedridden for 4 days before his death. The event was related not to the study drug but to the progression of the underlying disease.

Twelve other serious adverse events (SAEs) were reported during the study (Table 4): 6 SAEs in 6 patients in the 1 million cells/kg group, 5 SAEs in 4 patients in the 2 million cells/kg group, and 1 SAE in 1 patient in the SOC group. All these SAEs reported during the study were unrelated or remotely related to Stempeucel and resolved with residual effect or completely (urinary retention and gangrene) during the study period.

**Table 4:** Summary of serious adverse events

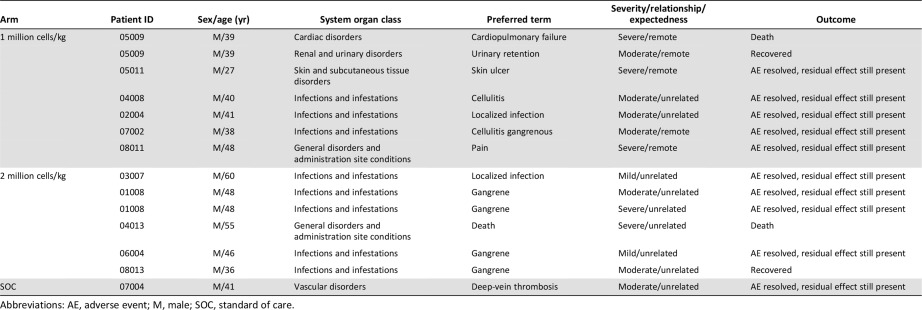

In our earlier phase I/II clinical study, immunological profile and lymphocyte profile were assessed at different time points in both the cell and placebo groups. These analyses revealed similar results in both groups, suggesting that the administered allogeneic cells did not adversely alter the immunological and lymphocytic profile [13].

### Efficacy Outcomes

All analyses were based on the modified intention‐to‐treat (MITT) principle (data from patients who had at least one postbaseline efficacy assessment). The study was statistically powered to demonstrate efficacy in the Stempeucel group.

#### Rest Pain

**Figure 3:**
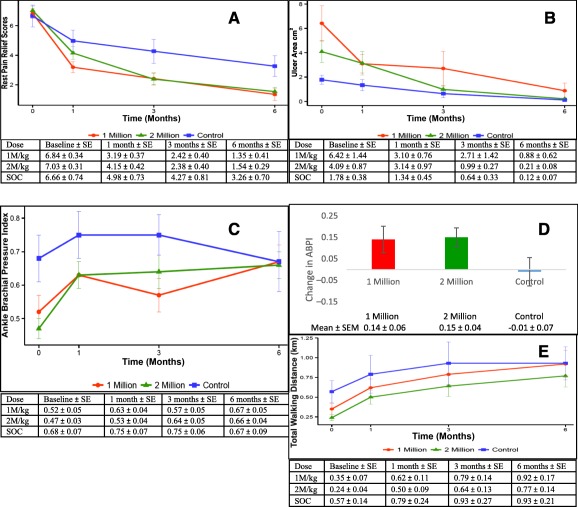
Assessment of clinical efficacy parameters at various time points. **(A):** Rest pain assessment: mean change from baseline with SE shown as error bars (modified intention‐to‐treat [MITT] population). Compared with the SOC group, the 1 million cells/kg group had a 0.23 (SE, 0.13)‐unit reduction in rest pain per month (*p* = .0815; 95% confidence interval [CI], −0.48 to 0.03); the 2 million cells/kg group had a 0.3 (SE, 0.13)‐unit reduction in rest pain per month compared with the SOC group (*p* = .0193; 95% CI, −0.55 to −0.05). **(B):** Ulcer area: mean change from baseline, with SE shown as error bars (MITT population). Compared with the SOC group, the group receiving 1 million cells/kg had a 2% (SE, 0.06%) decrease in ulcer size per month (*p* = .6967; 95% CI, 0.87–1.10); the group receiving 2 million cells/kg had an 11% (SE, 0.05%) decrease in ulcer size per month (*p* = .0253; 95% CI, 0.80–0.99). **(C):** ABPI: Mean change from baseline, with SE shown as error bars (MITT population). Compared with the SOC group, the 1 million cells/kg group had a 0.02 (SE, 0.01)‐unit increase in the ABPI per month (*p* = .1329; 95% CI, −0.01 to 0.05); the 2 million cells/kg group had a 0.03 (SE, 0.01)‐unit increase per month compared with the SOC group (*p* = .0132; 95% CI, 0.01–0.06). **(D):** ABPI: Mean change from baseline. The group receiving 1 million cells/kg and the group receiving 2 million cells/kg had a 0.14 and 0.15 increase in ABIP at 6‐month follow‐up compared with a decrease of 0.01 in the SOC arm. **(E):** Total waking distance: Mean change from baseline, with SE shown as error bars (MITT population). Compared with the SOC group, the 1 million cells/kg group had a 1.03 (SE, 0.01) times greater total walking distance per month (*p* = .0231; 95% CI, 1–1.06); the 2 million cells/kg group had a 1.03 (SE, 0.02) times greater total walking distance per month (*p* = .0577; 95% CI, 1–1.07). Abbreviations: ABPI, ankle brachial pressure index; 1M/kg, 1 million cells/kg body weight; 2M/kg, 2 million cells/kg body weight; SOC, standard of care.

The 1 million cells/kg group had a 0.23 (SE, 0.13)‐unit reduction in rest pain per month (*p* = .0815; 95% confidence interval, −0.48 to 0.03) as compared with the SOC group. However, the 2 million cells/kg group had a 0.3 (SE, 0.13)‐unit reduction in rest pain per month compared with the SOC group (*p* = .0193; 95% CI, −0.55 to −0.05) (Fig. 3A).

#### Ulcer Healing

The 1 million cells/kg group had a 2% (SE, 0.06%) decrease in ulcer size per month (*p* = .6967; 95% CI, 0.87–1.10) compared with the SOC group. The 2 million cells/kg group had an 11% (SE, 0.05) decrease in ulcer size per month (*p* = .0253; 95% CI, 0.80–0.99) (Fig. 3B).

#### Ankle Brachial Pressure Index

The 1 million cells/kg dose group had a 0.02 (SE, 0.01)‐unit increase in the ABPI per month when compared with the SOC group (*p* = .1329; 95% CI, −0.01 to 0.05). The maximum benefit was observed in the 2 million cells/kg group, which showed a 0.03 (SE, 0.01)‐unit increase in ABPI per month compared with the SOC group (*p* = .0132; 95% CI, 0.01–0.06) (Fig. 3C). A mean increase in ABPI at 6‐month follow‐up was seen in the cell groups compared with the SOC group (Fig. 3D).

#### Total Walking Distance

The 1 million cells/kg group had a 1.03 (SE, 0.01) times higher total walking distance per month when compared with the SOC group (*p* = .0231; 95% CI, 1–1.06). The 2 million cells/kg group had a 1.03 (SE, 0.02) times higher total walking distance per month compared with the SOC group (*p* = .0577; 95% CI, 1−1.07) (Fig. 3E).

#### Quality of Life

Compared with the SOC group, the 1 million cells/kg group had an increase in the QOL activity score of 0.16 (SE, 0.06) units per month (*p* = .0144; 95% CI, 0.03–0.28), an increase in QOL symptom domain of 0.18 (SE, 0.07) units per month (*p* = .0092; 95% CI, 0.05–0.32), and an increase in QOL pain domain scores of 0.18 (SE, 0.06) units per month (*p* = .0027; 95% CI, 0.06–0.3). Similarly, compared with the SOC group, the 2 million cells/kg group had an increase in the QOL activity domain score of 0.12 (SE, 0.07) units per month (*p* = .0529; 95% CI, 0–0.25), an increase in QOL symptom domain score of 0.15 (SE, 0.07) units per month (*p* = .0329; 95% CI, 0.01–0.3), and an increase in QOL pain scores of 0.13 (SE, 0.06) units per month (*p* = .0422; 95% CI, 0–0.25). However, QOL emotional domain, social domain, and total scores were not statistically significant in the cell groups.

#### MRA Analysis

**Figure 4:**
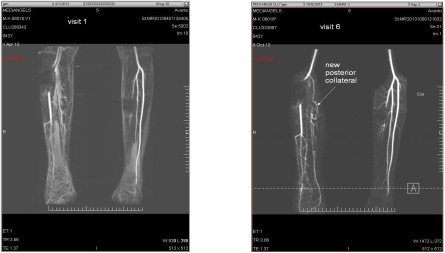
Magnetic resonance angiograms (left: baseline; right: 6 month) of one patient (ID number 08016) in the group receiving 2 million cells/kg body weight. Angiograms show overall increase in the vascularity of the affected limb, including development of new posterior collateral at 6‐month follow‐up after Stempeucel administration.

No significant difference was observed in the number of collateral vessels between the cell‐treated groups and the SOC group (*p* = .6524) at 6‐months follow‐up. However, more patients in the 2 million cells/kg group showed increased number of collateral vessels (45%) at 6‐month follow‐up compared with the SOC group (33.33%) or the 1 million cells/kg group (31.58%). This finding suggests that Stempeucel administration at the higher cell dose was capable of inducing collateral formation through proangiogenic activity (Fig. 4).

#### Total Amputation and Amputation‐Free Survival

Overall, 12 patients underwent amputations during the study period. Five patients (13.89%) were from the 1 million cells/kg group, 4 patients (11.114%) were from the 2 million cells/kg group, and 3 patients (16.67%) were from the SOC group. Amputation‐free survival at 6‐month follow‐up was 85.29%, 88.57%, and 82.35% in the 1 million cells/kg, 2 million cells/kg, and SOC groups, respectively. Hazard ratios were 0.85 (*p* = .8268) for the 1 million cells/kg versus the SOC group 0.64 (*p* = .5644) for the 2 million cells/kg versus the SOC group.

## Discussion

To our knowledge, this is the largest prospective, dose‐finding phase II study assessing the efficacy and safety of intramuscular administration of allogeneic human bone marrow‐derived MSC population (Stempeucel) in critical limb ischemia due to Buerger’s disease. Stempeucel injected intramuscularly in the gastrocnemius muscle and locally around the nonhealing ulcer resulted in significant reduction of rest pain and healing of ulcers in an accelerated fashion in the 2 million cells/kg treatment group compared with the SOC group.

The probable mechanism of action of Stempeucel in CLI is through secretion of growth factors, chemokines, and cytokines that are known for their angiogenesis, anti‐inflammatory, and immunomodulatory functions [20]. Stempeucel secretes angiogenic cytokines, such as vascular endothelial growth factor (VEGF), interleukin (IL)‐6, IL‐8, angiopoietin‐1, hepatocyte growth factor (HGF), keratinocyte growth factor, and insulin‐like growth factor‐1, and they are proven to be potently angiogenic [21]. Among all the factors secreted, VEGF has a unique feature compared with other growth factors in the regulation of new blood vessel formation with high degree of specificity for endothelial cells. VEGF treatment of cultured endothelial cells promotes cell survival, proliferation, migration, calcium influx, and branching morphogenesis [22]. The major immunomodulatory cytokines include prostaglandin 2, transforming growth factor‐β1, HGF, stromal cell‐derived factor‐1, nitrous oxide, indoleamine 2,3‐dioxygenase, IL‐4, IL‐6, IL‐10, IL‐1 receptor antagonist, and soluble tumor necrosis factor‐α receptor. These important factors play a key role in angiogenesis and act via the paracrine pathway [23,24]. These properties of Stempeucel are believed to be important to reinitiate blood flow by means of angiogenesis and arteriogenesis, therefore providing a rationale of therapeutic benefit in the treatment of Buerger’s disease.

In this study we had used BALB/c nude mice (OlaHsd‐Fox1nu) as an efficacy model for CLI. Both immunocompetent and immune‐compromised strains of mice have been used to determine the proangiogenic properties of various cell populations [17,25,26]. Because no animal model specific for Buerger’s disease is available, we chose to determine the preclinical efficacy of Stempeucel in a hind limb ischemia model induced by resection of the femoral artery in BALB/c nude mice [17]. In this report, we show that intramuscular administration of Stempeucel around the adductor muscle following femoral artery resection prevented foot necrosis and, in some cases, salvaged the limb.

The assessment of pain is challenging because it is a multifaceted expression of affective, cognitive, physical, sensory, behavioral, social‐cultural, and subjective feelings. Despite this diversity, the VAS pain scale is widely used in the literature and clinical practice. The VAS scale has a good validity and reliability [27] and is a simple and frequently used method to evaluate variations in pain intensity [28]. Cell‐based therapy trials [29, 30, 31, 32, 33] in general have reported high levels of pain relief. The Randomized‐Start, Placebo‐Controlled Pilot Trial (PROVASA) evaluated 40 patients with CLI due to peripheral arterial disease (PAD) (*n* = 32) or Buerger’s disease (*n* = 8). That multicenter, phase II, double‐blind study randomly assigned patients to receive intra‐arterial administration of BMMNCs or placebo. Cell therapy conferred a statistically significant advantage in terms of improved ulcer healing and reduced rest pain [34]. In our trial, we could see statistically significant reduction in rate of change in pain scores in patients exposed to 2 million cells/kg as compared with patients receiving SOC.

Ulcer healing is recommended as one of the important primary endpoints for clinical trials in patients with CLI at Fontaine stage IV [35]. Ulcer healing properties of BMMSCs were demonstrated by Dash et al. [36]. That study assessed the efficacy and feasibility of autologous BMMSCs in the treatment of chronic nonhealing ulcers (diabetic foot ulcers and ulcers in Buerger’s disease) of the lower extremities (*n* = 24). The cell group showed significant improvement in reduction in ulcer size compared with the control group at 12‐week follow‐up. Lu et al. compared autologous bone marrow mesenchymal stem cells with bone marrow‐derived mononuclear cells for treatment of diabetic critical limb ischemia and foot ulcer [37]. The ulcer healing rate of the BMMSC group was significantly higher than that of BMMNCs at 6 weeks after injection and reached 100% 4 weeks earlier than in the BMMNC group. Similarly, our phase II, dose‐finding study showed a statistically significant rate of ulcer healing (11% versus 2%) in the group receiving 2 million cells/kg compared with the group receiving SOC. We had also injected Stempeucel locally around the ulcer, which may have promoted ulcer healing through differentiation and angiogenesis [38,39].

There is an ongoing debate as to whether ABPI is a useful predictor for evaluating the long‐term efficiency of angiogenic therapy using stem cells. In a pooled analysis, Lawall et al. showed that autologous cell therapy can induce an increase in ABPI values of 0.1–0.2 points [40]. Another meta‐analysis revealed that ABPI improved from 0.46 ± 0.04 before cell therapy to 0.63 ± 0.04 after therapy (*p* = .011); in controlled trials of cell therapies, ABPI improved by 0.115 ± 0.060 (*p* = .054) [5]. In contrast, in the PROVASA trial, there were no significant differences in ABPI between the BMMNC and placebo groups [34], and the authors commented that changes in ABPI values did not correlate well with clinical endpoints, such as ulcer healing and limb salvage. However, Idei et al. showed that in Buerger’s disease, ABPI was significantly increased after 1 month of BMMNC implantation and remained high during the 3‐year follow‐up [4]. Similarly, our current study conducted in Buerger’s disease patients demonstrated a mean increase in ABPI of 0.15 points at 6‐month follow‐up in the 2 million cells/kg group, which is clinically beneficial to these patients. Hence, ABPI may be an important indirect indicator for evidence of angiogenesis, ulcer healing, and improvement in rest pain, at least in Buerger’s disease patients, because these patients have few cardiovascular risk factors and improvement in perfusion parameters may be maintained.

In patients with CLI due to atherosclerosis, the limb salvage outcome can be dismal [41]. However, this is not always true in the case of Buerger’s disease. The risks for amputation in Buerger’s disease are 25%, 38%, and 46% at 5‐, 10‐, and 20‐year follow‐up [42]. It is highly correlated with continued smoking and may reach up to 55% [43] in smokers. In one study, the 4‐year amputation‐free rates after BMMNC implantation were 48% in PAD patients and 95% in Buerger’s disease patients and 0% in control PAD patients and 6% in control Buerger’s disease patients [4]. In the current study, at 6‐month follow‐up the amputation‐free survival rates for the 1 million cells/kg, 2 million cells/kg, and SOC group were 85.29%, 88.57%, and 82.35%, respectively. Amputation‐free survival did not differ among the three groups at 6‐month follow‐up.

We conducted MRA after 6 months of Stempeucel administration to demonstrate new visible collateral vessels as evidence of improved blood flow. Lu et al. showed significantly increased collateral vessels (increased scores > 2) in the BMMSC group (*p* = .008) in diabetic CLI patients at 24‐week follow‐up [36]. The PROVASA trial revealed no significant differences in angiographically visible collateral vessels in the first 10 patients in the cell group [34]. Increased collateral vessels were seen in all 3 groups, and, importantly, best results (45%) were seen in the 2 million cells/kg group, although the difference did not show statistical significance. This may be due to underfilling of small collateral vessels or some collateral vessels being too small for identification by MRA as a result of the limited spatial resolution of magnetic resonance imaging [44].

Tumorigenesis, acute infusional toxicity, and infection are some concerns with use of stem cells. In a systematic review and meta‐analysis, Lalu et al. evaluated the safety data of different clinical trials using mesenchymal stem cells (MSCs) by intravascular delivery of MSCs in adult or mixed adult and pediatric population [45]. A total of 1,012 participants with various clinical conditions were included. There was a significant association between MSCs and transient fever that was not associated with long‐term sequelae. Importantly, the pooled analysis found no association between MSCs and tumor formation. In our study, a total of 47 AEs were reported in 28 patients in all dose groups. These AEs were assessed as remotely related or unrelated to Stempeucel; rather, they were assessed as related to the underlying disease. One event (pain at the site of Stempeucel administration) was assessed as probably related to Stempeucel and resolved within 1 month of follow‐up. Two deaths were reported during the study period and were remotely related, unlikely to be related, or unrelated to Stempeucel, as determined by the investigators.

This study used the GEE method for statistical evaluation of the data. This powerful technique has been used in longitudinal studies with repeated measurements, as seen in our study [46]. This method uses the correlation structure of outcomes as covariate and corrects for the dependence/correlation in the outcome measures. Advantages of this method are that it handles (a) the unbalanced time period/interval and the variability in the baseline as seen in our data, (b) repeated post‐outcome measures, and (c) missing data [46].

This study did have some limitations. It was a nonrandomized, open‐label study that may have been subject to potential bias in patient selection. Patients in the cell group had lower ABPI values and larger ulcers because investigators might have been biased to select more severe patients in the cell group. Despite this, patients recruited into the 2 million/kg group showed statistically significant improvement in both the primary endpoints and most secondary endpoints. Secondly, most patients in the trial were ex‐smokers, but urine cotinine was not tested to confirm their smoking status during the study. Nevertheless, the clinical study results presented here scientifically and clinically justifies the use of allogeneic BMMCS population obtained from healthy donors as a viable therapeutic option for Buerger’s disease patients.

## Conclusion

This phase II, open‐label nonrandomized study demonstrated the possible effects of Stempeucel in CLI due to Buerger’s disease. The patients in the 2 million cells/kg group showed clinical benefit in both the primary endpoints (rest pain relief and ulcer healing) and most secondary endpoints (improvement in total walking distance, ankle brachial pressure index, and quality of life). Hence, it is suggested that in this pathfinder study, a Stempeucel dose of 2 million cells/kg body weight may be the best dose in patients with critical limb ischemia due to Buerger’s disease, but further randomized controlled trials are required to confirm the exact dose. The next phase of the study is planned for a larger patient population to confirm both the efficacy and safety of Stempeucel.

## Author Contributions

P.K.G.: conception and design, collection and/or assembly of data, data analysis and interpretation, manuscript writing, final approval of manuscript; M.K., S. Desai, R.M., S. Dutta, U.S., R.R., A.D., and R.P.: provision of study material or patients, collection and/or assembly of data; A.C.: conception and design, collection and/or assembly of data, data analysis and interpretation, manuscript writing; L.J.: data analysis and interpretation; P.V. and R.N.S.: provision of study material or patients; P.K.V., C.T., and S.B.: collection and/or assembly of data; M.R.: performance of preclinical studies; A.S.M.: conception and design, collection and/or assembly of data, data analysis and interpretation.

## Disclosure of Potential Conflicts of Interest

P.K.G., A.C., P.V., P.K.V., R.N.S., C.T., M.R., S.B., and A.S.M. are employees of Stempeutics Research Pvt. Ltd. S. Dutta, U.S., and L.J., have compensated research funding. R.R. received research funding from Stempeutics Research Pvt. Ltd. The other authors indicated no potential conflicts of interest.
